# Prevalence of Hepatitis B virus between Qeshm Island people in 2013-2014, Iran

**Published:** 2015

**Authors:** A Kargar Kheirabad, E Elmira Jokari, MJ Sajjadi, H Gouklani

**Affiliations:** * Department of Virology, School of Public Health, Tehran University of Medical Sciences, Tehran, Iran,; ** Student Research Committee, Hormozgan University of Medical Sciences, Bandar Abbas, Iran,; *** Molecular Medicine Research Center, Hormozgan University of Medical Sciences, Bandar Abbas, Iran

**Keywords:** epidemiology, Hepatitis B, Qeshm

## Abstract

**Introduction:** About 1/ 3 of the world crowd (2 billion) suffers from HBV infection. 15 to 40% of Hepatitis B cases develop into chronic hepatitis, cirrhosis, and hepatocellular carcinoma (HCC). Considering the dangerous complication of the illness and the evidence that the prevalence is different in various areas of the country, this research was directed with the purpose of determining the currency of the Hepatitis B between Qeshm Island crowds.

**Method:** This cross-partial research was directed on 1500 cases. The sampling procedure was the stratify-cluster organization. Later creating the checklist, including the demographic information and risk factors, blood cases were formed. ELISA system evaluated the currency of HBsAg. At the end, the mathematical studies were conducted by applying the mathematical Plans for software of Social Sciences (SPSS) system issue 16.0. The information were investigated by Chi-square and detailed mathematical exams.

**Result:** The overall currency of HBsAg positivity was 1%, 0.8%, and 1.1% between male and female, individually. The middle age of members was 30.07 years old. Virus was more currency in married persons, students, lower than in 15-years-old educated people and persons who had a past of vaccination and transfusion. The currency of Hepatitis B in people who had a past of sex and substance infusion was zero. Finally, the finding of the research showed that none of the investigated factors was associated with the prevalence of HBsAg.

**Conclusion:** It appears that the currency of HBV virus in Qeshm is slightly lower than that of the public.

## Introduction

Hepatitis B virus (HBV) was discovered in 1966. Pursuant to the Organization of World Health (WHO), HBV-infected almost 1/ 3 of the world’s population (2 billion) [**1**], among whom more than 350000000 crowd global are chronically affected with the disease [**2**-**4**] and 75% of them live in Asia [**5**]. HBV virus is one of the deadliest diseases which points to the death of 0.5-1.2 million people annually. It was reported that 15-40% of HBV-infected cases would improve persistent hepatitis, cirrhosis and hepatocellular carcinoma (HCC), in whom the later plays a major pathogenic role worldwide [**6**] and accounts for 320000 deaths each year [**3**,**7**]. Hepatitis B prevalence varies in various sectors of the universe from 1 to 20%. Overall, approximately 45% of the global population lives in areas of hyper-endemic HBV prevalence where the amount of hepatitis B outside antigen (HBsAg) is reported in extra than 8 percentage in these areas. 43% of the world’s crowd alive in the intermediate-endemic regions with a prevalence of 2 to 7%; and 12% live in low-endemic areas of lower than 2% predominance of HBsAg [**7**-**9**]. In the hyper-endemic areas, the risk of infection with HBV is of more than 60% and the transfer mostly occurs prenatally from mother to baby. This value is between 20 to 60% in intermediate-endemic areas like Iran and can affect all ages. In the low-endemic regions, the risk of infection decreases to lower than 20% and limits to adults (World Health Organization). HBsAg, Hepatitis B e Ag (HBeAg) and antibodies against HBeAg are tested by ELISA The appearance of HBsAg in the plasma for six months or longer is indicative of chronic Hepatitis B infection [**10**].

The complications of chronic Hepatitis B include advancement to cirrhosis and liver failure, hepatocellular carcinoma and extra-hepatic diseases (polyarteritis nodosa, glomerulonephritis and leukocytoclastic vasculitis) [**11**]. HBV is transmitted prenatally or by percutaneous and mucous membrane exposures to infectious body fluids, such as serum, semen, and saliva [**12**].

The currency of HBsAg in Iran was described to be among 2.5% and 7.2% in 1979. In the 1980s, approximately 3% of the people was concerned, changing of a currency valuation of 1.7 percent in the Fars Zone to 5 percent in Sistan-Balouchestan Province [**13**-**15**]. The most common routes of communication considered are perinatal transmission and intravenous drug abuse [**16**].

There has been little work done to investigate the currency of HBV in Iran until now and it appears that the effect of this problem is underestimated. Given that the currency of HBV varied in various zones and based on what was said, the study investigates the prevalence of Hepatitis B among the inhabitants of the Qeshm Island.

## Method

During these two years (2013-2014) cross-sectional study, the statistical population included 130000 people of Qeshm Island. The sample size was calculated to be 1500, by applying the current formula:

$$n=\frac{z_{1-\frac{\alpha }{2}}^{2}p(1-p)}{d^{2}}$$

The island was divided into several regions in which a multistage group sampling chose participants from people who referred to health centers, hospitals, and health houses in different areas. Data were obtained by applying a checklist that was created based on related education and the experts' opinion. After the selection of persons, the study's goal was apparently talked with the members and demographic aspects and therapeutic records containing name, age, gender, job, level of education, history of sexual contact, history of vaccination, records of blood transmission and injecting drug use, were collected by using a checklist.

The addition measures include being a Qeshm citizen and giving approval. Participants with cardiovascular diseases, hypertension, rheumatoid fever, active infections, recent measles infection, mumps and IMN, history of malaria, toxoplasma, brucellosis, tuberculosis, HBs-Ag+, HCV-Ab+, HIV or its probability, gastrointestinal diseases such as ulcers, blood or sexual diseases, pregnancy, lactation, recent accident, receiving immunoglobulin during three months previous to the study, mental disorders, diabetes, thyroid disorders or patient in-cooperation, were deprived from the research. 

In this study, ten-milliliter venous blood cases were received from any person. Centrifugation divided serum, and cases were collected at -20°C. Serum cases were selected for HBsAg by ELISA applying a third production Kit (Biomeriex, Amsterdam). Positive samples (according to ELISA method) were referred for polymerase chain reaction (PCR) to establish the appearance of HBsAg. Patients with positive HBsAg in PCR were recognized as being infected with Hepatitis B. 2 images of the lab analysis results were provided. One of these was given to the members, and different was encoded, and the study followed an unsigned image.

Obtained information was listed in SPSS v.16 application and explained by applying detailed statistics (frequency, determine, percentages, and normal variation) and chi-square exam.

## Results

A whole character of 1500 people was included in this research, 511 men, and 989 women. HBsAg positivity of the serum samples were investigated by ELISA and the PCR method where 1% (11 females and 4 males) with the middle age of 30.07 and regular variation of 13.69 (P=0540) were HBsAg positive, and the remaining 99% (1485 persons) with the mean age of 32.63 and regular variation of 13.17 were HBsAg negative (P=0.340). HBV infection was only detected in lower than 15-year-old educated people (P=0.390). The largest amount of HBV prevalence was detected in the students (1.8%; 4 persons) while the others (workers, employees, and un-employees), were HBV negative (P= 0.510) (**Table 1**).

According to **Table 2**, one person had a history of substance infusion and 4 persons had the sex history, however, they were not infected (P=1.000). HBV virus currency was published to be 2.9% (2 persons) for those people with transfusion history, while it was 0.9% (13 persons) for the remaining people with no history of transfusion (P=370). Finally, the finding of this research showed that none of the investigated factors was related to the currency of HBsAg.

**Table 1:** Demographic characteristics of members

Variable	No.	HBsAg Positive	P-value
Sex			
Men	511	4 (0.8%)	0.540
Women	989	11 (1.1%)	
Education			
Less than 15 years	1344	15 (1.1%)	0.390
More than 15 years	156	0 (0%)	
Job			
Household	723	8 (1.1%)	0.510
Free	323	2 (0.6%)	
Health personnel	91	1 (1.1)	
Student	217	4 (1.8%)	
Employee	101	0	
Worker	16	0	
Un-employee	29	0	

**Table 2:** Risk factors associated with Hepatitis B surface antigens

Variable	No.	HBsAg Positive (%)	P-value
History of injecting drug use			
Yes	1	0%	1.000
No	1499	1%	
History of sexual contact			
Yes	4	0%	1.000
No	1496	1%	
Hepatitis B vac.			
Yes	489	1%	0.570
No	540	0.9%	
Unknown	471	1.1%	
Transfusion history			
Yes	70	2.9%	0.370
No	1430	0.9%	
Unknown	17	0%	

## Discussion

HBV virus is a health problem of global importance in which many people are involved worldwide [**17**,**18**]. Countries in the Middle East region are different concerning the currency of HBV. Kuwait and Bahrain can be classified as low-endemic countries, whereas Oman, Palestine, Yemen, Egypt, Jordan and Saudi Arabia are high-endemics [**19**]. Iran is recognized as a low- to intermediate- endemicity region about the currency of HBV.

The studies have shown that the currency of HBV is not the same in different parts of the country. In this current research, this value was estimated to 1% which is below than the whole national statistics (2.14%) [**20**]. Also, these results showed lower values than Todd et al. [**21**], McQuillan et al. [**22**], Shahin Merat et al. [**23**], Erden et al. [**24**], Abdolahi et al. [**25**], Salehi et al. [**26**], Moezzi et al. [**27**], Ansari-Moghaddam et al. [**28**] and Fathimoghaddam et al. [**29**] studies. Of course, the prevalence value was greater than the values estimated by Wasley et al. [**30**] and Keyvani et al. [**31**]. In Ghadir et al. study [**32**], the currency of HBsAg was published at 1.3%. These differences in prevalence could be because of social differences that lead to the vaccination of more people and the avoidance of high-risk sexual behaviors, etc.

The mean age of cases affected with HBV was 30.07 years. HBV prevalence in persons older than 50 years was greater than the younger’s’ in the works of Wasley et al. [**30**] and McQuillan’s et al. [**22**]. In the research of Erden et al. [**24**], the most of currency was observed in the ages between 21 and 40 years. Gogos et al. [**33**], Salehi et al. [**26**], and Ansari-Moghaddam et al. [**28**] showed that the HBV is more prevalent in persons older than 65 years.

In our study, the incidence of HBV in women was more than that of the men, which is consistent with Roshandel’s work [**34**], but changes from other investigations [**23**-**25**, **28**, **29**]. This difference is due to the greater amount of females employed in this study than males; and the outcome could change if the equality could change. 

The prevalence of HBV was only detected in those people with the education level of less than 15 years, where the amount of HBsAg was of approximately 1.1%. This outcome matched with the study of McQuillan et al. [**22**], in which the disease is more common in people with the literacy less than high-school. In Merat’s et al. [**23**] work, the currency of HBV in people with the literacy less than 12 years was 4%, while in others it was 3%. Ghadir [**32**] noted that the most of HBsAg could be found in the serum sample of illiterate people (2.95%) whereas people with academic degree showed the lowest amount of HBsAg. The reason for the lower prevalence in the well-educated people is their good awareness about the transmission ways of HBV and the avoidance of the high-risk sexual practices.

In this current research, the largest prevalence was recognized in the students while it was zero in the unemployed people. Failure to produce the entertainment possibilities and thus the tendency to the unhealthy entrainments such as addiction or risky sexual behaviors might cause the mentioned result. Gogos et al. [**33**] reported the largest amount of HBV prevalence in farmers. In opposition to our work, Erden et al. [**24**] and Ghadir et al. [**32**] have showed that the lowest rate is related to the students. Also, Taeri et al. [**35**] reported that 90% of the HBsAg positives were unemployed and mentioned that it could be due to their tendency to high-risk jobs such as selling sex and drug trafficking.

We did not see any HBV positivity in the serum sample of individuals with substance infusion history. In the study of Machado et al. [**36**], the currency of 0.4% was identified in those people and Moezzi’s research [**27**], this rate was 6%. Another work [**37**] showed the HBV rate of 1.9% and 9.7% for those with below than 1 year and more than 1 year history of drug injection, respectively. These variations could be because of the different economic, social, and cultural conditions, and also supportive care services and the used tools.

In this research, the HBV currency was 0 for those people with a sexual contact history which agreed with the Keyvani’s work [**31**]. McQuillan’s study [**22**] showed that the most of sex partners, the higher the prevalence of HBV there was, so that those persons with more than 50 sex partners showed a rate of 6.5%, and in Jahani et al. study [**38**], this rate was 2.5%. Todd [**21**] showed that the HBsAg prevalence for homosexual men was 4% and for heterosexual persons with the substance infusion it was 2%. The zero-rate in this research could be because of the religious beliefs (in Iran or other Islamic countries) which limit the great number of sex partners.

In the current research, the rate of infection in people with the past of transfusion (2.9%) was more than that of those with no transfusion (0.9%), which disagrees by the research of Jahani et al. [**38**]. In the researches of Salehi [**26**] and Fathimoghadam (3.17%) [**26**,**29**], the HBV prevalence was more than that in our result. Ghadir [**32**], Keyvani [**31**], and Moezzi [**27**] reported the rates of 1.63%, 1.88%, and 4%, respectively. In the Mirershadi’s research [**39**], it was shown that there was a direct relation between the times of bleeding and the HBV virus in the thalassemia patients; therefore that the amount of 8.3% was detected in those patients with more than 20 bleeding times. 

## Conclusion

The number of HBV prevalence in Qeshm and its suburbs was 1%; so it classified as a low endemicity area in Iran. In this research, the maximum rate of HBV infection was observed in the household women with literacy of below than fifteen years old and the past of transfusion or vaccination. Also, for the employed people, the highest rate of infection was estimated for the students.

**Acknowledgements**

This current research was the outcome of a general physician research. We would like to thank all professors, Qeshm residents, health organization officials, and Medical Sciences Hormozgan University who supported us through this research.
